# Association Between Increased Nuchal Translucency and Foetal CNS Abnormalities in Euploid Foetuses: Systematic Review and Meta-Analysis

**DOI:** 10.3390/diagnostics16091250

**Published:** 2026-04-22

**Authors:** Giula Mackina, Belen M. Ricci, Mirjam Moser, Christos Chatzakis, Kypros H. Nicolaides, Anastasija Arechvo

**Affiliations:** 1 Harris Birthright Research Centre, Fetal Medicine, King’s College Hospital, SE5 8BB London, UK; giula.mackina@gmail.com (G.M.); maria.ricci@nhs.net (B.M.R.); mirjam.moser@nhs.net (M.M.); cchatzakis@gmail.com (C.C.); kypros@fetalmedicine.com (K.H.N.); 2 Department of Women and Children’s Health, School of Life Course and Population Sciences, King’s College London, SE1 7EH London, UK

**Keywords:** first trimester, nuchal translucency, CNS, brain anomalies, neural tube defects

## Abstract

**Objective:** Increased nuchal translucency (NT) thickness at 10–14 weeks’ gestation is a well-established marker of chromosomal abnormalities, foetal structural defects, genetic syndromes, and foetal death; however, its association with foetal central nervous system (CNS) abnormalities has not been systematically evaluated. This study aimed to review and synthesise existing evidence on the relationship between first-trimester increased NT and prenatal ultrasound–detected foetal CNS abnormalities. **Methods:** A systematic literature search of MEDLINE, Embase, and CINAHL was conducted in accordance with PRISMA guidelines and registered in PROSPERO. Studies reporting increased NT in singleton pregnancies and structural abnormalities of the foetal CNS identified on prenatal ultrasound were included. Study selection, data extraction, and quality assessment were performed independently by two reviewers. **Results:** Twenty-three studies, including 15,592 euploid pregnancies with increased NT, met the inclusion criteria. Definitions of increased NT varied across studies, most commonly >95th centile or ≥3.5 mm. The pooled prevalence of CNS anomalies was 1.16% (95% CI 0.68–1.95; I^2^ = 80%). In three comparative studies including 6040 pregnancies with increased NT and 152,682 with normal NT, increased NT was associated with higher odds of CNS anomalies (OR 3.22, 95% CI 1.52–6.80; I^2^ = 74.1%). **Conclusions:** These findings suggest that euploid foetuses with increased NT may have a higher risk of CNS abnormalities.

## 1. Introduction

Increased nuchal translucency (NT) thickness at 10–14 weeks’ gestation is a well-established marker of chromosomal abnormalities, many foetal defects and genetic syndromes, as well as foetal death [1,2,3]. There is a clear association between increased NT and cardiac and extracardiac anomalies [4,5]. However, no studies have specifically evaluated the association between increased NT and the risk of structural abnormalities of the central nervous system (CNS). Early identification of potential CNS anomalies is clinically relevant, as it may influence prenatal surveillance, counselling, and pregnancy management. We hypothesised that euploid foetuses with increased NT have a higher risk of CNS abnormalities detectable on prenatal ultrasound compared with foetuses with normal NT measurements.

The objective of this study is to review the existing evidence on the association between first-trimester increased NT and CNS abnormalities identified on prenatal ultrasound in euploid foetuses, and to discuss the implications of this association in subsequent investigation and follow-up.

## 2. Methods

### 2.1. Literature Search and Study Selection

A systematic search of MEDLINE, Embase, and CINAHL Complete was performed to identify studies reporting an association between increased NT in singleton pregnancies at 10–14 weeks’ gestation and structural foetal CNS abnormalities detected on prenatal ultrasound at any gestational age. NT measurements in the included studies were performed during the first-trimester scan according to Nicolaides et al. [6]. CNS abnormalities were primarily diagnosed by prenatal ultrasound examination, with confirmation by follow-up imaging or postnatal evaluation where available. The search followed the PRISMA 2020 guidelines [7] and was registered in PROSPERO (CRD420251113963). The literature search was conducted in January 2026 without restriction on starting date and was limited to English and Spanish language publications. Full search strategies for all databases are provided in Appendix A.1.

The abstracts of citations were examined by two reviewers (G.M. and B.M.R.) to identify all potentially relevant articles, which were then examined in full-text form. Reference lists of relevant original and review articles were hand-searched for additional publications. Agreement about potential relevance was reached by consensus and by consultation with a third reviewer (A.A.). Exclusion criteria were case reports (fewer than five cases), review articles or guidelines, and those that did not distinguish between euploid and aneuploid pregnancies in order to eliminate the confounding effect of chromosomal abnormalities. Studies involving twin pregnancies were also excluded because NT discordance in monochorionic twins may reflect placental hemodynamic imbalance and early manifestations of twin-specific complications rather than structural foetal abnormalities. During the preparation of the illustrative material for this manuscript, the authors used ChatGPT (GPT-5.3; OpenAI).

### 2.2. Data Extraction and Quality Assessment

We used a standardised table to extract the following information from all of the included articles: first author(s), publication year, country, study population, data source, follow-up years, definitions of increased NT, sample size, and cases of CNS abnormality.

The quality of included studies was assessed using the Newcastle Ottawa Scale [8] as recommended by the Cochrane Non-Randomised Studies Methods Working Group. In this scale, each study is evaluated according to eight items categorised into three groups: the selection of the study groups, the comparability of the groups, and the ascertainment of the outcome. Each item was graded with a maximum score of one point, except for comparability, which allowed for two points. The total score ranged from 0 to 9 points, with higher scores indicating higher quality. Quality assessment was performed by two authors (G.M. and B.M.R.) independently. The two authors reviewed the tool and agreed on a method of implementation before their independent study assessments. The level of agreement between the two authors was calculated by another author (C.C.).

### 2.3. Statistical Analysis

Data from each study were extracted, and the number of events and total sample size were recorded. Study-specific proportions with corresponding 95% confidence intervals (CIs) were calculated, and pooled estimates were obtained, weighted according to the inverse variance of each study.

To account for the distributional properties of proportions and stabilise variances, individual study estimates were logit-transformed prior to pooling. Study-level CIs were calculated using the Clopper–Pearson exact binomial method. In studies reporting zero events, a continuity correction of 0.5 was applied [9].

Given the nonrandomized design of the included studies and the anticipated clinical and methodological heterogeneity (including variation in NT definitions and study populations), summary effect sizes were calculated using random-effects models with restricted maximum-likelihood (REML) estimation of between-study variance (τ^2^) [10].

The random-effects model assumes that the true underlying effect varies across studies and therefore incorporates both within-study and between-study variability, providing more conservative pooled estimates with wider confidence intervals. Common-effect models were calculated as sensitivity analyses.

Between-study heterogeneity was assessed using Cochran’s Q test and quantified with the I^2^ statistic. Subgroup analyses were performed according to the NT definition, and differences between subgroups were evaluated using a χ^2^ test for interaction. Forest plots were constructed to illustrate study-specific and pooled estimates.

To evaluate the association between increased NT and CNS anomalies, study-specific odds ratios (ORs) were pooled. Odds ratios were calculated using the Mantel–Haenszel method and combined using inverse-variance weighting under both common-effect and random-effects models, with REML estimation of τ^2^. Heterogeneity was assessed using Cochran’s Q and I^2^ statistics.

All analyses were performed using the meta package in R 4.5.3 (R Foundation for Statistical Computing, Vienna, Austria).

## 3. Results

**Figure 1:**
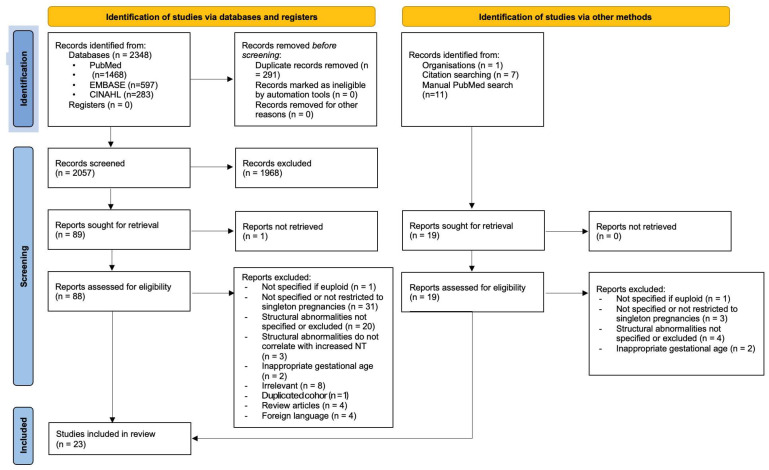
PRISMA flowchart of the inclusion process.

Study selection is summarised in the PRISMA flow diagram (Figure 1). A total of 2098 records were identified through database and hand searching. Of these, 1994 did not meet the inclusion criteria. 104 full-text articles were assessed for eligibility. Authors of 20 studies were contacted to clarify population characteristics, with six providing additional information. Following review, 81 studies were excluded because they were not restricted to singleton pregnancies, had overlapping data with other included cohorts, the cohort was not specified as euploid, did not describe foetal defects, or findings were not correlated with increased NT. A total of 23 studies (*N* = 15,592) reporting on euploid foetuses with increased NT were included in the final analysis [11,12,13,14,15,16,17,18,19,20,21,22,23,24,25,26,27,28,29,30,31,32,33]; 14 were retrospective cohort studies, and 9 were prospective. PRISMA checklist is available in Appendix A.2.

In eight studies the definition of high NT was >95th centile [15,17,18,23,25,29,31,32], in eight it was ≥3.5 mm [14,19,20,21,22,26,28,30], in two each it was >99th centile [17,24], ≥3 mm [11,33], and ≥4 mm [12,13], respectively, and in one it was ≥5.5 mm [27].

**Figure 2:**
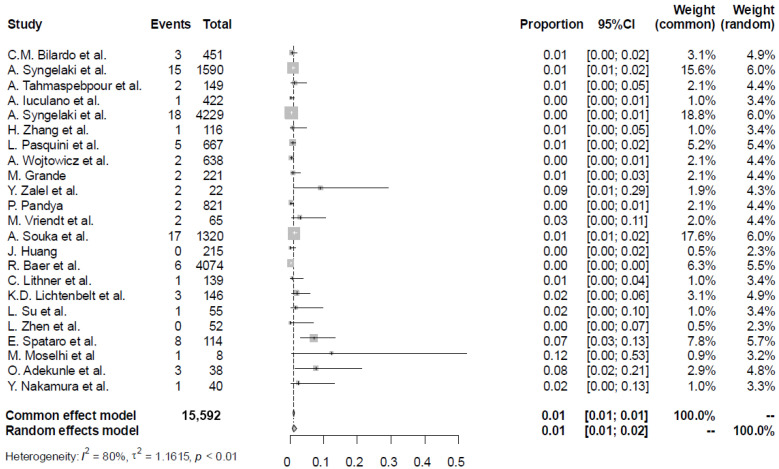
Forest plot of the CNS findings in the population of women with increased nuchal translucency [11,12,13,14,15,16,17,18,19,20,21,22,23,24,25,26,27,28,29,30,31,32,33].

In the combined data from all studies, there were 96 foetuses with CNS anomalies. The most common finding was ventriculomegaly seen in 17 cases, followed by 15 cases of anencephaly, 12 cases of spina bifida, 12 cases of abnormal posterior fossa including Dandy-Walker malformation, cerebellar hypoplasia and vermian agenesis, 11 cases of encephalocele, 11 cases of holoprosencephaly, seven cases of agenesis of corpus callosum, four cases of a complex brain abnormality, two cases of arachnoid cysts, and one case each of microcephaly, macrocephaly, polymicrogyria, craniosynostosis and occipital dermoid cyst. A summary of study characteristics and results is provided in Table 1. The overall pooled prevalence of CNS anomalies was 1.16% (95% CI, 0.68–1.95; I^2^ = 80%) (Figure 2).

**Figure 3:**
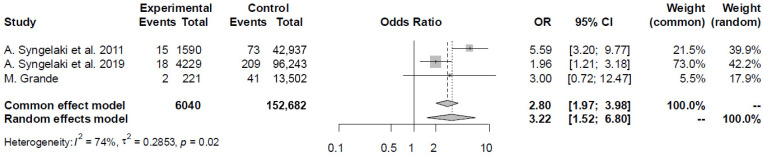
Forest plot of the CNS findings in the population of women with and without increased nuchal translucency [16,17,25].

Three studies compared the prevalence of CNS anomalies in pregnancies with high NT and in those with NT below the 95th centile (Figure S1). In the high NT group (*N* = 6040), there were 35 (0.6%) cases with CNS defects, and in those with normal NT (*N* = 152,682), there were 323 (0.2%) cases of CNS defects. Increased NT was associated with significantly higher odds of CNS anomalies (OR 3.22, 95% CI, 1.52–6.80; I^2^ = 74.1%) (Figure 3).

**Figure 4:**
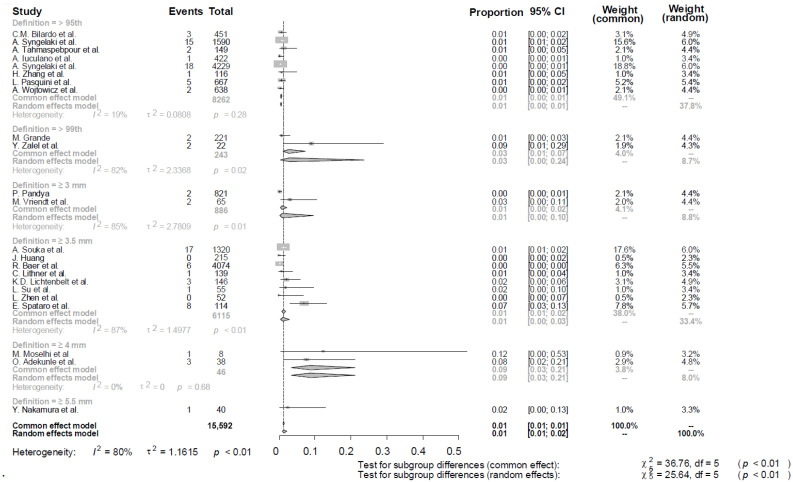
Forest plot of the CNS findings in the population of women with increased nuchal translucency, grouped by the different definitions used for the increased nuchal translucency [11,12,13,14,15,16,17,18,19,20,21,22,23,24,25,26,27,28,29,30,31,32,33].

When stratified according to the NT definition, significant differences were observed between subgroups (*p* ≤ 0.001 for subgroup differences, random-effects model). Studies defining increased NT as >95th centile demonstrated a pooled CNS anomaly prevalence of 0.62% (95% CI, 0.47–0.83), with low heterogeneity (I^2^ = 19%). For NT ≥ 3.5 mm, the pooled prevalence was 1.29% (95% CI, 0.94–1.78; I^2^ = 87%). For NT ≥ 3 mm, the pooled prevalence was 0.86% (95% CI, 0.32–2.27; I^2^ = 85%). For NT > 99th centile, the pooled prevalence was 2.79% (95% CI, 1.04–7.27; I^2^ = 82%). For NT ≥ 4 mm, the pooled prevalence was 8.84% (95% CI, 3.35–21.31; I^2^ = 0%). For NT ≥ 5.5 mm, the pooled prevalence was 2.50% (95% CI, 0.35–15.73; I^2^ = not applicable), derived by a single study (Figure 4).

### Quality of Studies

A total of 23 studies were assessed using the Newcastle-Ottawa Scale (NOS) (Appendix A.3). Overall methodological quality was predominantly low. Final NOS scores ranged from 5 to 9 out of a maximum of 9 points.

Seven studies (30.4%) [16,17,18,20,25,30,33] were rated as good quality (NOS score ≥ 7), including three studies [16,20,25] achieving the maximum score of 9/9. The remaining 16 studies (69.6%) were classified as poor quality (scores 4–6).

Most studies demonstrated adequate performance in the selection and outcome domains, with several achieving the maximum score for selection (4/4) and outcome assessment (3/3). However, comparability was the main methodological limitation. Most studies (20/23, 86.6%) received one point in the comparability domain, indicating limited adjustment for potential confounders. Only three studies [16,20,25] achieved two points for comparability.

## 4. Discussion

### 4.1. Main Findings and Interpretation of Results

In this systematic review, we synthesised evidence from 23 studies, including 15,592 euploid singleton pregnancies, evaluating the relationship between increased NT and foetal CNS anomalies. Despite heterogeneity in study design and NT thresholds, increased NT thickness was associated with a higher risk of CNS abnormalities, compared with euploid foetuses with NT below the 95th centile. The wide range of CNS pathologies identified suggests that enlarged NT may not be linked to a single developmental pathway but may reflect diverse underlying mechanisms.

**Figure 5:**
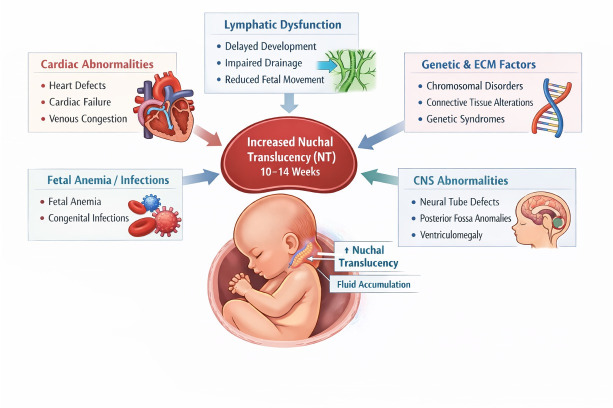
Mechanisms involved in increased nuchal translucency include delayed or abnormal development of the lymphatic system, impaired lymphatic drainage, fetal anemia, congenital infections, chromosomal disorders, cardiac dysfunction and abnormally developing brain. Illustrative material created by ChatGPT (GPT-5.3; OpenAI).

Increased NT at 10–14 weeks has been linked to several biological mechanisms, including cardiac failure due to structural abnormalities of the heart and great vessels, venous congestion in the head and neck from mechanical compression, and alterations in the extracellular matrix associated with chromosomal or genetic disorders. Additional contributors include delayed or abnormal lymphatic development, impaired lymphatic drainage secondary to reduced foetal movements, foetal anaemia, and congenital infections leading to anaemia or cardiac dysfunction [4] (Figure 5). However, the pathogenesis of increased NT in cases of neural tube defects and other brain abnormalities in euploid foetuses remains poorly understood. It is important to note that the purpose of this systematic review was to synthesise the available evidence on the association between increased first-trimester NT and CNS abnormalities in euploid foetuses. Currently, there is no well-established pathophysiological pathway explaining how NT enlargement leads to CNS anomalies. As such, our findings are observational and focus on quantifying the association rather than proposing a causal or testable biological model.

Congenital structural anomalies of the CNS occur in approximately 2–3 per 1000 total births [34]. They pose diagnostic challenges because accurate detection often requires advanced imaging techniques, such as detailed foetal neurosonography and foetal brain magnetic resonance imaging, as well as longitudinal assessment throughout pregnancy.

Ventriculomegaly is the most common foetal CNS abnormality [35], a finding that was likewise reflected in our cohort, where it accounted for 17.7% of all CNS anomalies. In contrast, Syngelaki et al. [25] included only cases of severe ventriculomegaly (≥15 mm), which likely contributed to its lower reported prevalence. Ventriculomegaly is often a progressive condition that becomes apparent in the second or third trimester, highlighting the importance of continued surveillance in foetuses with increased NT. Additionally, high rates of termination of pregnancy in certain studies, such as that by Zalel et al. [24], may have limited the identification of brain abnormalities that develop later in gestation. Postnatal data further support this association, demonstrating a threefold increased risk of congenital hydrocephalus in foetuses with NT measurements at or above the 95th percentile [20].

Posterior fossa abnormalities represent another major diagnostic challenge, as a wide range of conditions, from normal variants to severe malformations, can share similar sonographic features. Several studies [14,15,16,18,24,30,32,33] have reported foetuses with increased NT and posterior fossa abnormalities such as Dandy–Walker malformation, cerebellar hypoplasia, and vermian hypoplasia. However, the biological link between increased NT and these abnormalities remains unclear.

Holoprosencephaly, a genetically and phenotypically heterogeneous disorder, has been reported in association with trisomy 13, trisomy 18, and increased NT, and has also been described in cases of triploidy. However, studies [14,16] reported several euploid foetuses with increased NT and holoprosencephaly. Beyond chromosomal abnormalities, holoprosencephaly may result from single-gene disorders affecting key developmental pathways and leading to complex syndromes, highlighting the need for further genetic evaluation in foetuses with holoprosencephaly and increased NT.

Foetal neurosonography is an evolving field of prenatal imaging that continues to advance in parallel with technological progress. Detailed neurosonographic evaluation enables comprehensive assessment of foetal brain anatomy and facilitates the detection of subtle abnormalities [36,37]. The development of high-resolution ultrasound systems has made it possible to evaluate the foetal brain at earlier stages of pregnancy and to correlate findings with embryological development, thereby improving visualisation of fine anatomical structures and enhancing our understanding of both normal and abnormal brain development [38].

In particular, novel ultrasound markers detectable as early as the first trimester allow reliable prediction of abnormalities traditionally identified during the second-trimester scan, including ventriculomegaly and posterior fossa malformations [38,39]. However, a study of over 100,000 singleton pregnancies demonstrated that, even when the second-trimester anomaly ultrasound is normal, a substantial proportion of foetal brain defects are first detected in the third trimester [40]. Furthermore, as the most critical period of foetal cortical development occurs between 22 and 32 weeks of gestation, several recent studies have advocated for additional neurodevelopmental assessment beyond the routine anatomical examination [41].

Future studies should prioritise standardised definitions of increased NT, systematic use of detailed foetal neurosonography, and incorporation of postnatal and long-term neurodevelopmental follow-up. Such approaches will improve risk stratification, enhance prenatal counselling, and optimise management strategies for pregnancies complicated by increased NT. Future mechanistic studies will be required to explore potential developmental or molecular pathways underlying these observations.

### 4.2. Clinical Implications

Increased NT may be used as an early phenotypic marker for a broader range of CNS malformations. Therefore, increased NT should prompt counselling regarding elevated overall structural risk, with a significant CNS component, even when the foetal karyotype is normal. A detailed first-trimester scan [2], followed by a comprehensive second-trimester anatomy scan with focused CNS assessment [42,43], including midline structures and posterior fossa, should be recommended.

### 4.3. Strengths and Limitations

A major strength of this study is that it represents the first systematic review specifically focused on CNS outcomes in euploid foetuses with increased NT, addressing an important gap in the existing literature. The review was conducted in accordance with PRISMA guidelines, with strict study selection criteria and independent assessment by two independent reviewers to minimise selection bias.

This study has several limitations. First, the overall sample size is relatively small, and there was substantial heterogeneity among the included studies (I^2^ = 80%), likely reflecting differences in NT definitions, ultrasound techniques, gestational ages, and study populations. Although we performed subgroup analyses by NT thresholds, these do not fully account for the observed variability.

Second, none of the included studies had as a primary objective the assessment of foetal brain abnormalities; most focused on overall outcomes of foetuses with increased NT. Consequently, detailed neurosonography and serial examinations were not consistently applied, which may have led to underdetection of some CNS abnormalities.

Third, the number of comparative studies including foetuses with normal NT measurements was limited; only three studies provided control groups, restricting the strength of conclusions regarding the relative risk of CNS anomalies.

Fourth, potential detection bias is present, as most studies relied solely on prenatal ultrasound findings, with systematic postnatal confirmation inconsistently available. Some CNS abnormalities may only become apparent later in pregnancy or after birth, and the true incidence may be further underestimated as some pregnancies did not progress into the third trimester due to intrauterine foetal demise or termination of pregnancy.

We also acknowledge that single-gene disorders could represent a potential confounder in euploid foetuses; however, most studies did not perform genetic testing beyond conventional karyotyping. Additional limitations include potential selection bias from referral populations and the lack of standardised long-term neurodevelopmental follow-up.

These limitations underscore the need for well-designed prospective studies with standardised imaging protocols, systematic genetic testing, and long-term neurodevelopmental follow-up to better elucidate the relationship between increased NT and CNS abnormalities.

## 5. Conclusions

In conclusion, the available evidence suggests that euploid foetuses with increased NT may have a higher incidence of CNS abnormalities compared with foetuses with normal NT measurements. These findings highlight the importance of careful and continued evaluation of the foetal brain in pregnancies with increased NT, even in the absence of chromosomal abnormalities. Targeted neurosonographic assessment and follow-up imaging later in gestation may be valuable for the early detection of subtle CNS anomalies. However, the current evidence is limited by heterogeneity among studies, variations in NT definitions, and the lack of standardised follow-up and long-term outcome data. Further large prospective studies with standardised neurosonographic protocols and comprehensive postnatal follow-up are needed to better clarify the relationship between increased NT and foetal CNS development.

## Figures and Tables

**Table 1:** Summary of included studies reporting on euploid foetuses with increased NT and incidence of CNS abnormalities.

First Author, Year	NT Cut-Off	Total	Defects	Specified Defect
Pandya, 1995 [11]	≥3 mm	821	2	Anencephaly, holoprosencephaly
Moselhi, 1996 [12]	≥4 mm	8	1	Spina bifida
Adekunle, 1999 [13]	≥4 mm	38	3	Anencephaly, encephalocele, macrocephaly
Souka, 2001 [14]	≥3.5 mm	1320	17	Anencephaly, spina bifida, encephalocele, holoprosencephaly, ventriculomegaly, Dandy Walker malformation
Bilardo, 2007 [15]	>95th	451	3	Anencephaly, spina bifida, Dandy-Walker malformation
Syngelaki, 2011 [16]	>95th	1590	15	Acrania, spina bifida, holoprosencephaly, agenesis of corpus callosum, vermian agenesis
Grande, 2012 [17]	>99th	221	2	Acrania, encephalocele
Tahmasebpour, 2012 [18]	>95th	149	2	Ventriculomegaly, Dandy-Walker malformation
Huang et al., 2014 [19]	≥3.5 mm	215	0	None
Baer et al., 2014 [20]	≥3.5 mm	4074	6	Hydrocephalus, encephalocele, brain deformity
Lithner et al., 2015 [21]	≥3.5 mm	139	1	CNS defect (not specified)
Lichtenbelt et al., 2015 [22]	≥3.5 mm	146	3	Ventriculomegaly, holoprosencephaly, complex brain
Iuculano et al., 2016 [23]	>95th	422	1	Arachnoid cyst
Zalel et al., 2017 [24]	>99th	22	2	Holoprosencephaly, vermian agenesis
Syngelaki et al., 2019 [25]	>95th	4229	18	Holoprosencephaly, encephalocele, spina bifida, agenesis of corpus callosum, arachnoid cyst, craniosynostosis, occipital dermoid cyst
Su et al., 2019 [26]	≥3.5 mm	55	1	Ventriculomegaly
Nakamura et al., 2020 [27]	≥5.5 mm	40	1	Encephalocele
Zhen et al., 2022 [28]	≥3.5 mm	52	0	None
Zhang et al., 2023 [29]	>95th	116	1	Ventriculomegaly
Spataro et al., 2023 [30]	≥3.5 mm	114	8	Ventriculomegaly, agenesis of corpus callosum, vermis hypoplasia
Pasquini et al., 2023 [31]	>95th	667	5	Holoprosencephaly, agenesis of corpus callosum, polymicrogyria, complex brain
Wojtowicz et al., 2024 [32]	>95th	638	2	Microcephaly, cerebellar hypoplasia
Vriendt et al., 2025 [33]	≥3 mm	65	2	Agenesis of the corpus callosum, posterior fossa abnormality
Total		15,592	96	

## Data Availability

The data presented in this study are available on request from the corresponding author.

## Supplementary Materials

The following supporting information can be downloaded at: https://www.mdpi.com/article/10.3390/diagnostics16091250/s1, Figure S1: Forest plot of the CNS findings in the population of women with increased nuchal translucency.

## Appendix A

### Appendix A.1. Search Strategies

**PubMed (1946–present)**

1. ((“pregnancy trimester, first”[MeSH Terms]) OR (“foetus”[MeSH Terms])) OR (First trimester[Title/Abstract] OR first-trimester[Title/Abstract] OR 1st trimester[Title/Abstract] OR Gestational week[Title/Abstract] OR gestation*[Title/Abstract] OR foetus[Title/Abstract] OR foetal[Title/Abstract] OR foetal[Title/Abstract] OR foetus[Title/Abstract])
2. (((“nuchal translucency measurement”[MeSH Terms]) OR (Nuchal translucency[Title/Abstract] OR NT[Title/Abstract]))) OR ((nuchal[Title/Abstract] AND (ultrasound[Title/Abstract] OR ultrasonography[Title/Abstract] OR scan*[Title/Abstract])))
3. (((((“brain”[MeSH Terms]) OR (“brain diseases”[MeSH Terms])) OR (“central nervous system diseases”[MeSH Terms])) OR (“nervous system malformations”[MeSH Terms])) OR (“chromosome aberrations”[MeSH Terms])) OR (Brain*[Title/Abstract] OR (brain[Title/Abstract] AND (pathology*[Title/Abstract] OR defect*[Title/Abstract] OR anomal*[Title/Abstract] OR malformation*[Title/Abstract] OR adverse[Title/Abstract])) OR CNS[Title/Abstract] OR “central nervous system”[Title/Abstract] OR “neurological complication*”[Title/Abstract])
4. 1, 2, and 3

**Embase (1947–present)**

1. ‘first trimester pregnancy’/exp OR ‘foetus’/exp OR ‘first trimester’:ab,ti OR ‘first-trimester 1st trimester’:ab,ti OR ‘gestational week’:ab,ti OR gestation*:ab,ti OR foetus:ab,ti OR foetal:ab,ti OR foetal:ab,ti OR foetus:ab,ti
2. ‘nuchal translucency measurement’/exp OR ‘nuchal translucency’:ab,ti OR nt:ab,ti OR (nuchal:ab,ti AND (ultrasound:ab,ti OR ultrasonography:ab,ti OR scan*:ab,ti))
3. ‘brain disease’/exp OR ‘brain’/exp OR ‘central nervous system disease’/exp OR ‘nervous system malformation’/exp OR ‘chromosome aberration’/exp OR brain*:ab,ti OR (brain:ab,ti AND (pathology*:ab,ti OR defect*:ab,ti OR anomal*:ab,ti OR malformation*:ab,ti OR adverse:ab,ti)) OR cns:ab,ti OR ‘central nervous system’:ab,ti OR ‘neurological complication*’:ab,ti
4. 1, 2, and 3
5. 4 and [embase]/lim not ([embase]/lim and [medline]/lim)
6. 5 and (‘article’/it or ‘review’/it)

**CINAHLComplete (EBSCOHost, inception to present)**

S1. (MH “Pregnancy Trimester, First”)
S2. (MH “Foetus+”)
S3. TI (First trimester OR first-trimester 1st trimester OR Gestational week OR gestation* OR foetus OR foetal OR foetal OR foetus) OR AB (First trimester OR first-trimester 1st trimester OR Gestational week OR gestation* OR foetus OR foetal OR foetal OR foetus)
S4. S1 OR S2 OR S3
S5. (MH “Nuchal Translucency Measurement”)
S6. TI (Nuchal translucency OR NT) OR AB (Nuchal translucency OR NT) OR TI ((nuchal AND (ultrasound OR ultrasonography OR scan*)) OR AB ((nuchal AND (ultrasound OR ultrasonography OR scan*))
S7. S5 OR S6
S8. (MH “Brain+”)
S9. (MH “Brain Diseases+”)
S10. (MH “Central Nervous System Diseases+”)
S11. (MH “Nervous System Abnormalities+”)
S12. (MH “Chromosome Aberrations+”)
S13. TI (Brain* OR (brain AND (pathology* OR defect* OR anomal* OR malformation* OR adverse)) OR CNS OR “central nervous system” OR “neurological complication*”) OR AB (Brain* OR (brain AND (pathology* OR defect* OR anomal* OR malformation* OR adverse)) OR CNS OR “central nervous system” OR “neurological complication*”)
S14. S8 OR S9 OR S10 OR S11 OR S12 OR S13
S15. S4 AND S7 AND S14

### Appendix A.2. PRISMA Checklist

**Table A1:** PRISMA checklist.

Section and Topic	Item #	Checklist Item	Location Where Item Is Reported
**Title**			
Title	1	Identify the report as a systematic review.	Page 1 (Title)
**Abstract**			
Abstract	2	See the PRISMA 2020 for Abstracts checklist.	Page 1 (Abstract)
**Introduction**			
Rationale	3	Describe the rationale for the review in the context of existing knowledge.	Page 1 (Introduction, paragraphs 1–2)
Objectives	4	Provide an explicit statement of the objective(s) or question(s) the review addresses.	Page 2 (Introduction, final paragraph)
**Methods**			
Eligibility criteria	5	Specify the inclusion and exclusion criteria for the review and how studies were grouped for the syntheses.	Page 2 (Methods–Literature search and study selection)
Information sources	6	Specify all databases, registers, websites, organisations, reference lists, and other sources searched or consulted to identify studies. Specify the date when each source was last searched or consulted.	Page 2 (Methods–Literature search and study selection)
Search strategy	7	Present the full search strategies for all databases, registers, and websites, including any filters and limits used.	Appendix A.2
Selection process	8	Specify the methods used to decide whether a study met the inclusion criteria of the review, including how many reviewers screened each record and each report retrieved, whether they worked independently, and, if applicable, details of automation tools used in the process.	Page 2 (Methods–Literature search and study selection)
Data collection process	9	Specify the methods used to collect data from reports, including how many reviewers collected data from each report, whether they worked independently, any processes for obtaining or confirming data from study investigators, and, if applicable, details of automation tools used in the process.	Page 2 (Methods–Data extraction and quality assessment)
Data items	10a	List and define all outcomes for which data were sought. Specify whether all results that were compatible with each outcome domain in each study were sought (e.g., for all measures, time points, analyses), and if not, the methods used to decide which results to collect.	Page 2 (Methods–Data extraction)
	10b	List and define all other variables for which data were sought (e.g., participant and intervention characteristics, funding sources). Describe any assumptions made about any missing or unclear information.	Page 2 (Methods–Data extraction)
Study risk of bias assessment	11	Specify the methods used to assess risk of bias in the included studies, including details of the tool(s) used, how many reviewers assessed each study, and whether they worked independently, and if applicable, details of automation tools used in the process.	Page 2 (Methods–Data extraction and quality assessment)
Effect measures	12	Specify for each outcome the effect measure(s) (e.g., risk ratio, mean difference) used in the synthesis or presentation of results.	Page 3–4 (Methods–Statistical analysis)
Synthesis methods	13a	Describe the processes used to decide which studies were eligible for each synthesis (e.g., tabulating the study intervention characteristics and comparing against the planned groups for each synthesis (item #5)).	Page 3–4 (Methods–Study selection and data extraction)
	13b	Describe any methods required to prepare the data for presentation or synthesis, such as handling of missing summary statistics or data conversions.	Page 3–4 (Methods–Statistical analysis)
	13c	Describe any methods used to tabulate or visually display the results of individual studies and syntheses.	Page 4 (Methods–Statistical analysis; Forest plots)
	13d	Describe any methods used to synthesise results and provide a rationale for the choice(s). If meta-analysis was performed, describe the model(s), method(s) to identify the presence and extent of statistical heterogeneity, and software package(s) used.	Page 3–4 (Methods–Statistical analysis)
	13e	Describe any methods used to explore possible causes of heterogeneity among study results (e.g., subgroup analysis, meta-regression).	Page 4 (Methods–Statistical analysis; subgroup analyses)
	13f	Describe any sensitivity analyses conducted to assess the robustness of the synthesised results.	Page 4 (Methods–Statistical analysis)
Reporting bias assessment	14	Describe any methods used to assess the risk of bias due to missing results in a synthesis (arising from reporting biases).	Page 2 (Methods–quality assessment)
Certainty assessment	15	Describe any methods used to assess certainty (or confidence) in the body of evidence for an outcome.	Page 2 (Methods–quality assessment)
**Results**			
Study selection	16a	Describe the results of the search and selection process, from the number of records identified in the search to the number of studies included in the review, ideally using a flow diagram.	Page 4, 10 (Results–Study selection; Figure 1)
	16b	Cite studies that might appear to meet the inclusion criteria, but which were excluded, and explain why they were excluded.	Page 4, 10 (Results–Study selection)
Study characteristics	17	Cite each included study and present its characteristics.	Page 4 (Results; Table 1)
Risk of bias in studies	18	Present assessments of risk of bias for each included study.	Page 6, 14 (Results–Quality of studies; Appendix A.3)
Results of individual studies	19	For all outcomes, present, for each study: (a) summary statistics for each group (where appropriate) and (b) an effect estimate and its precision (e.g., confidence/credible interval), ideally using structured tables or plots.	Page 5, 6 (Results; Table 1, Figure 2, Figure 3 and Figure 4)
Results of syntheses	20a	For each synthesis, briefly summarise the characteristics and risk of bias among contributing studies.	Page 6 (Results–Quality of studies)
	20b	Present the results of all statistical syntheses conducted. If meta-analysis was done, present for each the summary estimate and its precision (e.g., confidence/credible interval) and measures of statistical heterogeneity. If comparing groups, describe the direction of the effect.	Page 5, 6 (Results; Figure 2, Figure 3 and Figure 4)
	20c	Present the results of all investigations of possible causes of heterogeneity among study results.	Page 5, 6 (Results; subgroup analyses)
	20d	Present results of all sensitivity analyses conducted to assess the robustness of the synthesised results.	Not applicable
Reporting biases	21	Present assessments of risk of bias due to missing results (arising from reporting biases) for each synthesis assessed.	Page 6 (Results quality of studies)
Certainty of evidence	22	Present assessments of certainty (or confidence) in the body of evidence for each outcome assessed.	Page 5, 6 (Results)
**Discussion**			
Discussion	23a	Provide a general interpretation of the results in the context of other evidence.	Page 7–9 (Discussion–Main findings)
	23b	Discuss any limitations of the evidence included in the review.	Page 8 (Discussion–Strengths and limitations)
	23c	Discuss any limitations of the review processes used.	Page 8 (Discussion–Strengths and limitations)
	23d	Discuss implications of the results for practice, policy, and future research.	Page 8 (Discussion–Clinical implications and future research)
**Other information**			
Registration and protocol	24a	Provide registration information for the review, including the register name and registration number, or state that the review was not registered.	Page 2 (Methods–PROSPERO registration)
	24b	Indicate where the review protocol can be accessed, or state that a protocol was not prepared.	Page 2 (Methods)
	24c	Describe and explain any amendments to information provided at registration or in the protocol.	No amendments
Support	25	Describe sources of financial or non-financial support for the review, and the role of the funders or sponsors in the review.	Page 1 (Abstract), Page 9 (Funding)
Competing interests	26	Declare any competing interests of review authors.	Page 9 (Competing interests)
Availability of data, code, and other materials	27	Report which of the following are publicly available and where they can be found: template data collection forms; data extracted from included studies; data used for all analyses; analytic code; any other materials used in the review.	Data extracted from included studies

### Appendix A.3. Newcastle Ottawa Scale Quality Assessment

**Table A2:** Quality assessment.

Author	Selection	Comparability	Outcome	Final Score	Final Score
Pandya, 1995 [11]	2	1	1	4/9	Poor
Moselhi, 1996 [12]	2	1	1	4/9	Poor
Adekunle, 1999 [13]	2	1	1	4/9	Poor
Souka, 2001 [14]	2	1	3	6/9	Fair
Bilardo, 2007 [15]	2	1	3	6/9	Fair
Syngelaki, 2011 [16]	4	2	3	9/9	Good
Grande, 2012 [17]	4	1	3	8/9	Good
Tahmasebpour, 2012 [18]	3	1	2	6/9	Good
Huang et al., 2014 [19]	2	1	2	5/9	Fair
Baer et al., 2014 [20]	4	2	3	9/9	Good
Lithner et al., 2015 [21]	2	1	2	5/9	Fair
Lichtenbelt et al., 2015 [22]	3	1	1	5/9	Poor
Iuculano et al., 2016 [23]	2	1	1	4/9	Poor
Zalel et al., 2017 [24]	2	1	2	5/9	Fair
Syngelaki et al., 2019 [25]	4	2	3	9/9	Good
Su et al., 2019 [26]	3	1	1	5/9	Poor
Nakamura et al., 2020 [27]	2	1	1	4/9	Poor
Zhen et al., 2022 [28]	2	1	2	5/9	Fair
Zhang et al., 2023 [29]	3	1	2	6/9	Fair
Spataro et al., 2023 [30]	3	1	3	7/9	Good
Pasquini et al., 2023 [31]	4	1	1	6/9	Poor
Wojtowicz et al., 2024 [32]	3	1	1	5/9	Poor
Vriendt et al., 2025 [33]	3	1	3	8/9	Good
