# Supplementary figures and images for: Association Between Increased Nuchal Translucency and Foetal CNS Abnormalities in Euploid Foetuses: Systematic Review and Meta-Analysis
Source: Diagnostics (Basel). 2026 Apr 22;16(9):1250. doi: 10.3390/diagnostics16091250 (PMC13163124; doi:10.3390/diagnostics16091250)

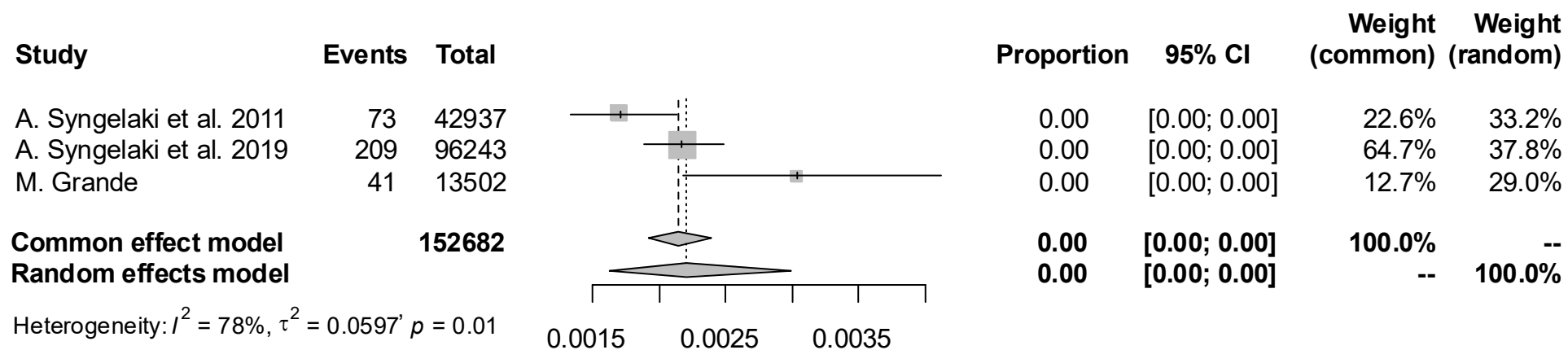

Figure S1.

Supplement: Supplementary file 1 [file diagnostics-16-01250-s001.zip › diagnostics-4204081-supplementary.pdf]
